# RNA Editing in Mitochondrial *Trans*-Introns Is Required for Splicing

**DOI:** 10.1371/journal.pone.0052644

**Published:** 2012-12-20

**Authors:** Jean-Claude Farré, Cindy Aknin, Alejandro Araya, Benoît Castandet

**Affiliations:** 1 UMR5234 Microbiologie Fondamentale et Pathologie, Centre National de la Recherche Scientifique and Université Bordeaux-Segalen, Bordeaux, France; 2 Institut de Biologie Végétale Moléculaire, UMR1332 Biologie du Fruit et Pathologie, Centre INRA de Bordeaux, Vilenave d'Ornon, France; NIGMS, NIH, United States of America

## Abstract

In plant mitochondria, gene expression of translatable mRNAs is a complex process with two critical steps, RNA editing and splicing. We studied the role of RNA editing on non-coding regions of the *mat-r-nad1e-nad5c* transcript from wheat mitochondria. This RNA contains two *trans*-introns, 3'-*nad1-I4* and 3'-*nad5-I2*, involved in different *trans-*splicing events, ensuring the association of *nad1d*-*nad1e* and *nad5b*-*nad5c* exons from *nad1* and *nad5* mRNAs respectively. The C-to-U editing changes studied here affect homologous positions on 3'-*nad1-I4* and 3'-*nad5-I2*. It is proposed that these base changes are necessary to place an Adenosine residue in a bulging conformation characteristic of domain VI (D6) from group II introns. In this work, we investigated the role of RNA editing events on 3'-*nad1-I4* and 3'-*nad5-I2* in the *trans*-splicing process using *in vivo* and *in organello* approaches. When the branched intermediates formed during the splicing process were analyzed, the C residues from D6 intron domains from 3'-*nad1-I4* and 3'-*nad5-I2* were found changed to U, suggesting that RNA editing of these residues could be mandatory for splicing. This assumption was tested by expressing recombinant *mat-r*-*nad1e* transgenes introduced into mitochondria by electroporation. Mutation of the editing target residue dramatically affected *trans*-splicing. Interestingly, the exon joining efficiency was not recovered by compensatory mutations, suggesting that the role of RNA editing is not confined to the restoration of the secondary structure of domain D6 of the intron. Our results strongly support the hypothesis that RNA editing in *trans*-introns precedes maturation, and is required for the splicing reaction. In addition, this is the first report using an *in organello* approach to study the *trans*-splicing process, opening the way to future studies of this peculiar mechanism.

## Introduction

In plant mitochondria, the production of the steady state pool of mRNAs ready for translation is a complex process. Following their transcription initiated from multiple promoters [1], [2], RNAs undergo several processing steps, the most critical being RNA editing and splicing [3], [4].

RNA editing proceeds by base specific deamination of cytosine into uracil [5]. These modifications mainly occur in coding regions and affect the amino acid sequence of the protein product [6], [7]. RNA editing is therefore an essential step to ensure the production of functional proteins [8], [9]. While the role of RNA editing in mRNA coding regions is easily explained by the effect on the protein product, its function in non-coding regions still remains controversial.

Group II introns, thought to be the ancestor of the spliceosomal introns and retroelements [10], [11], are the predominant intron type in plant mitochondria [3]. They are characterized by a secondary structure formed by six stem-loop domains (D1 to D6) arranged around a central wheel folded in a complex tertiary architecture [12], [13]. Based on structural predictions, RNA editing is proposed to restore mispaired residues in highly conserved stems [14]–[16]. An analogous situation has recently been reported for plant mitochondria group I introns [17]. These observations led to the hypothesis that RNA editing in introns may play a role in the splicing reaction.

Using a yeast heterologous splicing model, it was proposed that RNA editing is a prerequisite for splicing [18], however, *in vivo* analyses of splicing intermediates indicated the contrary [19]. Recently, Castandet *et al*. reported that editing and splicing can be connected through the modification of a single C-to-U change in domain I (D1) of the *rps10* mRNA intron [20].

Aside from the *cis*-introns described above, plant mitochondrial genomes contain several scattered genes requiring *trans*-splicing to produce translational competent mRNAs [3]. In wheat, the *nad1* gene is expressed from five independent transcripts through one *cis*- and three *trans*-splicing events [21] and the *nad5* mRNA is generated by two *cis*-splicing and two *trans*-splicing events from three independent transcripts [22]. Interestingly, one transcript, *mat-r-nad1e-nad5c,* is a common substrate for two different *trans-*splicing processes ensuring the linkage of nad1d/e and nad5b/c exons. In both cases the intron discontinuity is located at the variable domain IV (D4) in the canonical secondary structure of group II introns [12].

**Figure 1:**
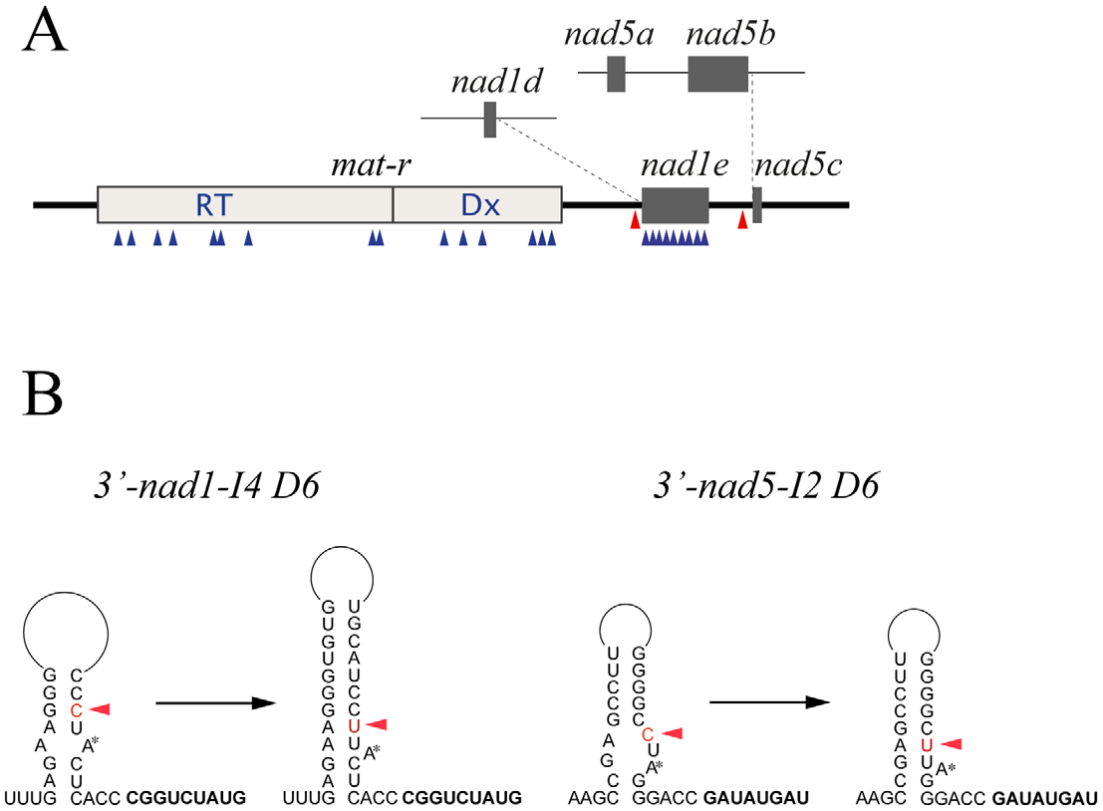
The *mat r-nad1e-nad5c* cotranscript from wheat mitochondria. (A) Scheme of the *mat r-nad1e-nad5c* RNA and the *trans*-splicing counterparts. Gray squares represent the fifth and the third exons of *nad1* and *nad5* respectively on the same molecule associated to the maturase-reverse transcriptase ORF (*mat-r*) [21], [24]. Blue arrowheads indicate the residues edited in the coding regions of the transcript. Red arrowheads indicate the edited residues in non-coding regions. Dotted lines indicate the splicing junctions of *nad1e* and *nad5c* exons with *nad1d* and *nad5b* exons. (B) The domain 6 of both, *nad1-I4* and *nad5-I2,* group II *trans*-introns is depicted as stem-loop structure. The most stable conformations are presented for the non-edited and edited mRNA. A red arrow indicates the edited residue.

Previous studies investigating the expression of the *mat-r-nad1e-nad5c* locus showed two C-to-U changes on domain VI (D6) of the respective *trans*-introns [23], [24] (Figure 1A). Although the D6 region within the 3′-region of *nad1-I4* and *nad5-I2* introns shows low sequence identity, they can be folded in an analogous stem-loop structure. Interestingly, both RNA editing events increase the stability of the D6 stem-loop, placing an adenosine residue in a bulge [24]. This conformation is characteristic of the lariat branch point, which is crucial for splicing of group II introns [25], [26]. The 2′-OH of the bulging adenosine acts as the nucleophile in the first transesterification step during splicing [27]. Consequently, it is reasonable to assume that RNA editing in *trans*-introns may play a role in the splicing reaction for *nad1e* and *nad5c.*

So far, no autocatalytic processing of group II introns from plant mitochondria have been reported, suggesting that these elements have evolved with a protein-assisted mode of RNA maturation. Recent evidence showed that multiple proteins in the PPR gene family participate in plant mitochondrial RNA splicing [28]–[30]. This situation makes it difficult to study the role of intronic structural elements in the splicing process. To circumvent this problem, we introduced *nad1e*-containing gene constructs into isolated mitochondria by electroporation [31]. This approach allows us to study the role of RNA editing on *nad1-I4* intron. Here, we show for the first time the editing status of these non-coding residues in splicing intermediates, and study the fate of a transgene bearing the *nad1e* exon in the *trans*-splicing reaction with endogenous *nad1* partners. Our results strongly support the hypothesis that RNA editing in *trans*-introns is involved in the splicing reaction.

## Materials and Methods

The vectors used in this work were based on *pCOX2Ta* [31], they contain the inverted repeat region from the wheat *cob* gene (*Ir-cob*) (accession no. AF337547). This region is the target for specific priming of chimeric splicing products in PCR analysis. The mutants were obtained with the QuickChange® Site-Directed Mutagenesis kit (Stratagene) under the conditions indicated by the furnisher. Restriction fragments and RT-PCR products were purified with the GFX PCR DNA and Gel Band Purification Kit (GE Healthcare). PCR products were cloned into the pGEM-T vector (Promega) using the TA cloning method.

### Assembling *nad1e* Constructs used for Electroporation Experiments

Wheat genomic DNA was used as template to obtain the region containing either the complete *mat-r* ORF, (*Mat1*) or the C-terminal domain Dx (*SDx1*), linked to the exon *nad1e* by PCR. Mat1 was obtained using primers Sac-MatR combined to SmaI-ex5nad1 and SDx1 was PCR amplified using primers Sac-Dx combined to Spe-ex5nad1 (see File S1). PCR products were purified using the Wizard® SV Gel and PCR Clean-Up System (Promega). Details on the regions of the *mat-r-nad1e-nad5c* locus used in the constructs are indicated in File S2.

PCR products were incubated with Sac1 and Sma1 endonucleases to obtain the Mat1 construct and with SacI and SpeI endonucleases for the SDx1 construct. PCR digestion products were ligated to the pCOX2Ta vector [31] digested with the same enzymes. The Sac1 digestion of the pCOX2Ta vector results in the loss of *cox2* in the recombinant plasmid. To obtain the constructs with the *cox2* promoter upstream of Mat1 or SDx1 sequences, the PCR primers Nsi-MatR and Nsi-Dx were used with primers SmaI-ex5nad1 and Spe-ex5nad1, respectively. The PCR products were digested with Nsi1 and Sma1, or Nsi1 and Sac1, and ligated to the pCOX2Ta vector digested with the same enzymes. The strategy used generates a *Dx* gene producing a transcript with 52 additional residues at the 3′ non-coding region, linking *nad1e* to the *IR-cob* terminator sequence, compared to the RNA from *Mat1.*

### Mitochondria Purification

Wheat mitochondria were prepared as previously described [31]. Mitochondria purified by centrifugation on a sucrose gradient were resuspended in 0.33 M sucrose. The protein content was determined using the Bio-Rad Protein Assay (Bio-Rad Laboratories, Inc.), adjusted to a concentration of 20 mg/ml, and used immediately in electroporation experiments.

### Mitochondria Electroporation

Electroporation was carried out using 1 mg of mitochondrial proteins and 2 µg of recombinant plasmid [31]. After the electroporation pulse, mitochondria were incubated for 18 h at 25°C with constant shaking (130 rpm) in a reaction mixture containing 0.33 M mannitol, 90 mM KCl, 10 mM MgCl_2_, 12 mM Tricine (pH 7.2), 5 mM KH_2_PO_4_, 1.2 mM EGTA, 10 mM sodium succinate, 1 mM GTP, 2 mM ADP, 0.15 mM CTP and 0.15 mM UTP, 2 mM dithiothreitol, and 1 mg/ml fatty acid-free BSA. Mitochondria were recovered by centrifugation at 15000×g for 15 min at 4°C. RNA was purified by extraction with 800 µl Trizol® reagent (Invitrogen) according to the supplier′s protocol.

### RT-PCR

Isolation and amplification of transgene RNA: one microgram of nucleic acids obtained after Trizol® treatment of the electroporated mitochondria was digested with 2 U of DNase I Amplification grade (Invitrogen) for 15 min at 25°C. cDNA synthesis was performed with 200 units of Superscript II RT (Invitrogen) using 100 ng of random hexamers (Promega). The PCR amplifications were performed with Advantage 2 polymerase mix (Clontech) using primers specific to the wheat *cob* gene (*Ir-cob*) and to the endogenous *nad1* transcript. The PCR parameters used were: 95°C for 2 min, 20 cycles at 95°C for 30 s, 64°C for 1 min and 68°C for 2 min, and finally 68°C for 10 min. If necessary, 2µl of this PCR product were then used in two subsequent nested PCR reactions with the same parameters except for annealing temperatures: 55°C and 51°C for PCR 2 and 3 respectively.

**Figure 2:**
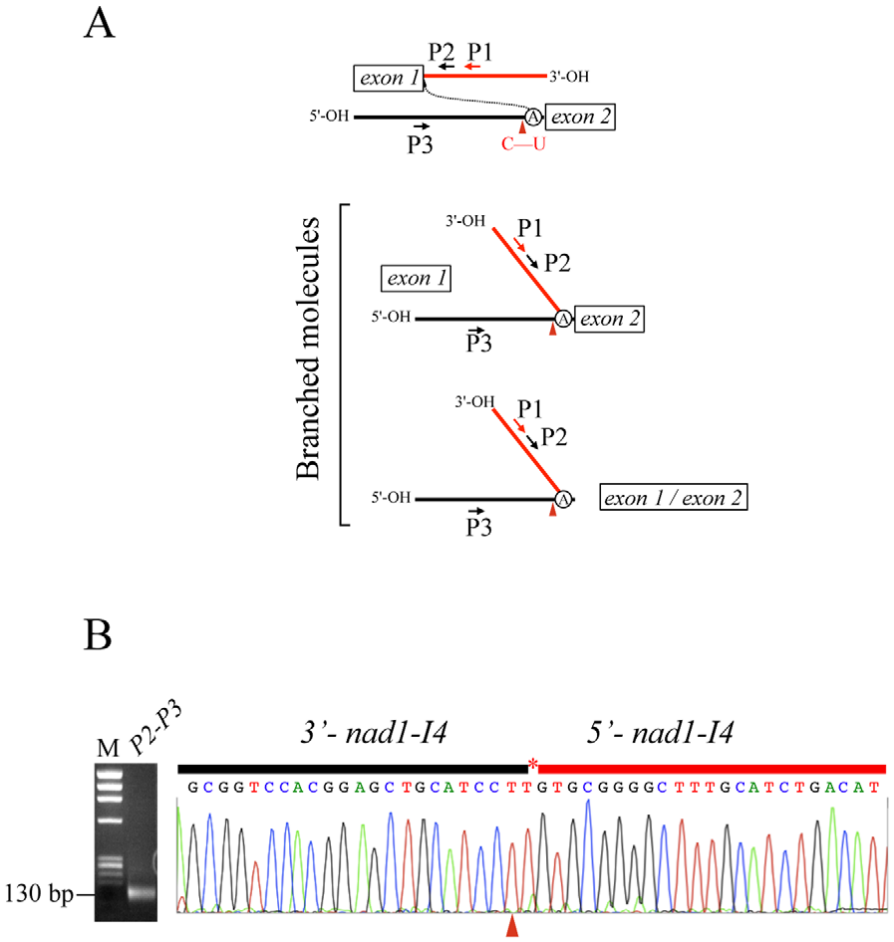
RNA editing in trans-splicing *nad1e* branched molecules. (A) Scheme of the branched splicing intermediate formed during *trans*-splicing. The position of the primers used to isolate the splicing intermediate are indicated by red and black arrows. Dotted lines indicate the nucleophilic attack of the 2′OH from the bulged A of domain D6 to obtain the 2′-5′-branched intermediate (B) Gel electrophoresis of the PCR products obtained by combining primers P2a and P3a (*nad1d/e*). The sequence of primers used to isolate *nad1e* and *nad5c* splicing intermediates is indicated in the File S1. The electropherograms of selected cloned PCR product is shown. Red arrowheads indicate the edited U generated by the conversion of the genome encoded C residue. The asterisk signals the A residue at the branching point; this residue is missing in the sequence electropherogram. M, PhiX174 DNA/HaeIII (Promega) molecular weight markers.

**Figure 3:**
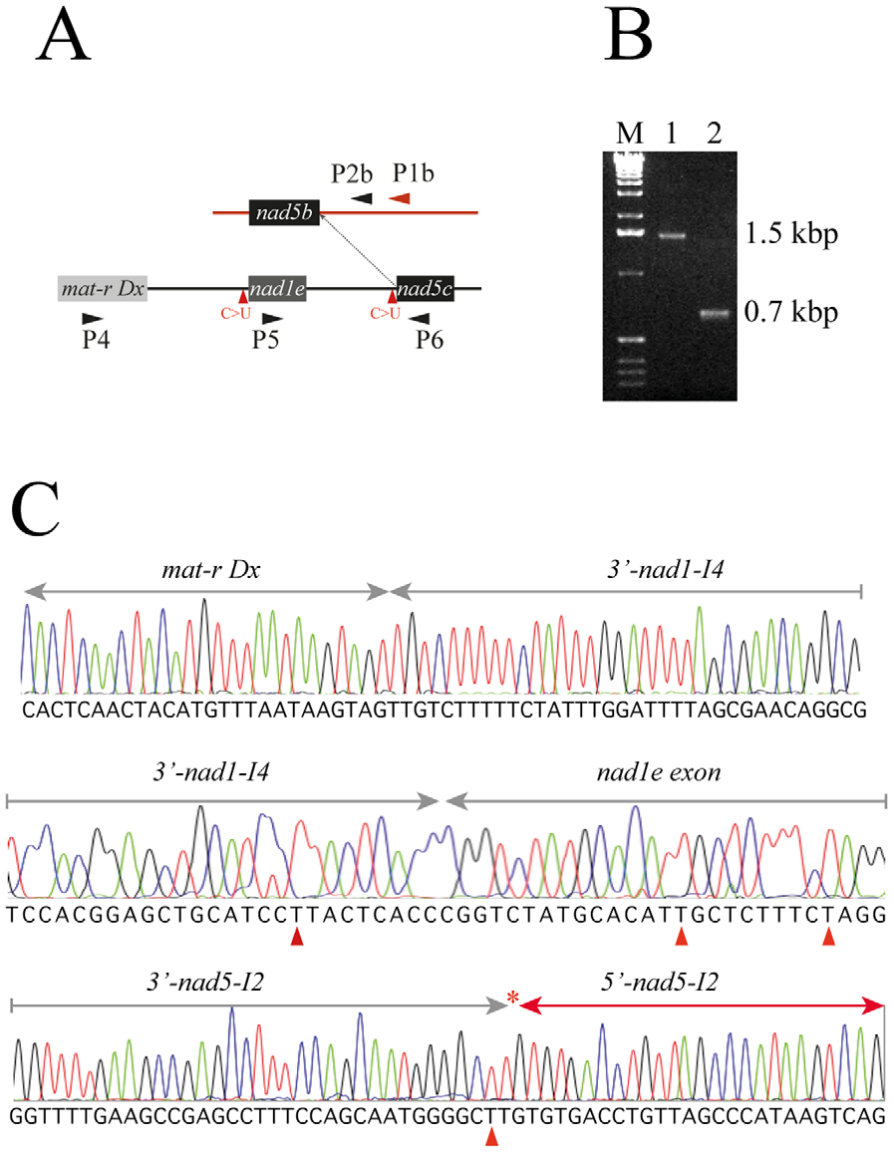
RNA editing in trans-splicing *nad5c* branched molecules. (A) Scheme of the *nad5b* and *mat-r-nad1e*-*nad5c* transcripts. The dimension of boxes do not represents the actual size of the exons. The primers used for branched splicing intermediate analysis are indicated by arrowheads. (B) Agarose gel electrophoresis analysis of RT-PCR products from the *nad5c* branched structure. cDNA was synthesized with primer P1b, followed by PCR amplification using primers P2b and P4 (lane 1) or primers P2b and P5 (lane 2). The primer P4 is specific of the maturase Dx domain and P5 is specific of exon *nad1e*. M, molecular weight markers. (C) Selected parts of the electropherogram of a 1.5 kbp cloned PCR product are shown. The sequence starting from the maturase domain Dx and the beginning of the 3′ half of the *nad1-I4* intron (upper panel), the end of the 3′ half of the *nad1e* intron and the *nad1e* exon (middle panel) and the *nad5c* branched splicing intermediate (lower panel) are shown. Red arrowheads show the position of edited residues.

Isolation and amplification of branched intermediates: RNA purification and cDNA synthesis was performed as described above. The reverse transcription was performed using specific primers P1a or P1b complementary to the 5′ region of the introns linked to exons *nad1d* and *nad5b*, respectively (Figure 2A and 3A). Primers P2a and P2b were used for nested PCR on 2 µl of cDNA, combined with either primers P3a or P3b. In both cases, P1 and P2 primers direct the DNA synthesis to the 5′-end of the half-transcript while the P3 primers, which are complementary to the sense strand, initiate DNA synthesis in a divergent direction. Moreover, P2 and P3 primer binding sites are on separate molecules. Consequently, P2 combined with P3 primers do not generate PCR products on mtDNA or cDNA, but only on the branched molecules. P2 and P3 primers were designed to produce a short PCR product of around 130 bp for both intermediates. Details of the primers used are indicated in File S1. In this study, only RNA preparations devoid of DNA contamination where used. Controls were performed by PCR amplification on control samples with no reverse transcriptase.

### Quantification of RNA Splicing

RT-PCR products were separated on agarose gel containing SYBR ®Safe DNA gel stain (Invitrogen) and run under standard electrophoresis conditions. The efficiency of splicing was determined from the fluorescence of SYBR Safe intercalated in DNA issued from precursor and mature molecules using a CCD camera coupled to a PC computer. Images obtained were scanned using ImageJ64 software (http://rsb.info.nih.gov/ij/).

### Quantification of RNA Editing

To determine the profile and rate of C-to-U conversions in RT-PCR products, the precursor and mature PCR bands were excised from the agarose gel after separation by electrophoresis. Bands were purified with the GFX PCR DNA and Gel Band Purification Kit (GE Healthcare). The purified fragments were ligated into pGEM-T easy vector as described by the manufacturer (Promega). Cloned PCR products were sequenced with the BigDye® Terminator Cycle Sequencing Kit v 1.1 (Applied Biosystems). Sequences analyses were performed at the Genotyping and Sequencing Facility of Université Victor Segalen-Bordeaux 2.

## Results

### The Branched Intermediates from *nad1d/e* and *nad5b/c trans*-splicing Present the C Target Residue Edited into U

The C-to-U change of both *nad1e-I4* and *nad5c-I2 trans*-introns in the *mat-r-nad1e-nad5c* co-transcript may affect the D6 stem-loop structure of the introns (Figure 1). The co-transcript is the substrate of two separate *trans*-splicing events allowing the connection between *nad1d* with *nad1e* exons and *nad5b* with *nad5c* exons. To assess whether this RNA editing is a prerequisite for splicing, we analyzed the branched structures of *nad1d-nad1e* and *nad5b*-*nad5c* resulting from the first transesterification reaction. The strategy used is shown in Figures 2A and 3A and detailed in the Methods section. Only the covalent branched molecules, resulting after the first transesterification steps during splicing, generated a PCR product using this approach. Combining P2 to P3 primers a PCR product of around 130 bp was obtained corresponding to branched molecules (Figure 2B), indicating the presence of *trans*-splicing intermediates in the steady state RNA pool. Similar Y-shaped intermediates resulting from *nad5b/nad5c* splicing process were obtained using analogous, P1b, P2b and P3b primers (not shown), and with primer P2b combined with P4 or P5 primers (Figure 3A. Primers used are detailed in File S1. The identity of the PCR products was confirmed by sequence analysis, showing that the short PCR molecules link both *trans*-intron halves by the predicted 2′-5′ phosphodiester bond at the bulging A (missing on electropherograms) (Figures 2B and 3C).

To verify the editing status of the C target located two residues upstream of the branching point in the D6 region, the PCR products were cloned and sequenced. All the branched molecules analyzed (24 clones for *nad1e* and 36 clones for *nad5c*) presented a U residue at the D6 editing site (Table 1). Similarly, the editing status of both C residues in the *mat-r-nad1e-nad5c* precursor was analyzed using primers P4 and P6 (see Figure 3A). Different to the branched intermediates, only about half of the clones analyzed were edited at the D6 C-target residue (Table 1). We note that some of the clones presented short insertions of 3 to 8 nucleotides after the branching point, an event previously described [32]–[34], indicating some inaccuracy in the first transesterification step. Nonetheless, all of these molecules also had the D6 C-target edited.

**Table 1:** Editing status of *nad1e* and *nad5c* intermediates.

Transcript	edited molecules (N° clones)
*nad1e* precursor	50% (10)
*nad1e* branched intermediate	100% (24)
*nad5c* precursor	40% (10)
*nad5c* branched intermediate	100% (36)

The PCR products from branched intermediates obtained with primers P2a and P3a specific for *nad1e* and primers P2b and P3b specific for *nad5c* were purified from agarose gel and cloned into pGEM-T, subsequently, selected clones were sequenced on both strands.

### The *nad5c* Exon can be Engaged in Splicing without the Loss of the Accompanying *mat-r* Dx Domain and the *nad1e* Exon

To verify if exons *nad5c* and *nad1e,* expressed on the same transcript, are a common substrate for two different *trans*-splicing reactions, we analyzed the presence of both exons in the *nad5c* branched splicing intermediate. For this purpose, PCR amplifications were performed on cDNA synthesized with primer P1b using primers P2b and either P4 or P5. Primers P4 and P5 are located on the C-terminal section of the *mat-r* ORF (Dx) and the *nad1e* exon, respectively (Figure 3A). We observed the expected 1523 and 663 bp products (Figure 3B), indicating that the first transesterification step linking the exon *nad5b* to *nad5c* may occur before splicing of *nad1d/e* exons. The PCR product obtained with P2b and P4 shows the editing status of both D6 regions on the same molecule in one sequence run. Parts of the branched intermediate sequence from the 1.5 kbp PCR product are depicted in Figure 3C. All samples analyzed presented both *nad1-I4* and *nad5-I2* D6 C residues changed to U. Moreover, editing sites found in coding regions, six on the domain Dx and nine on exon *nad1e* [24], were also edited, indicating that the this process occurs previously or is concomitant to RNA processing.

### The *nad1e* Transgene is a Substrate for Trans-splicing in Electroporated Mitochondria

**Figure 4:**
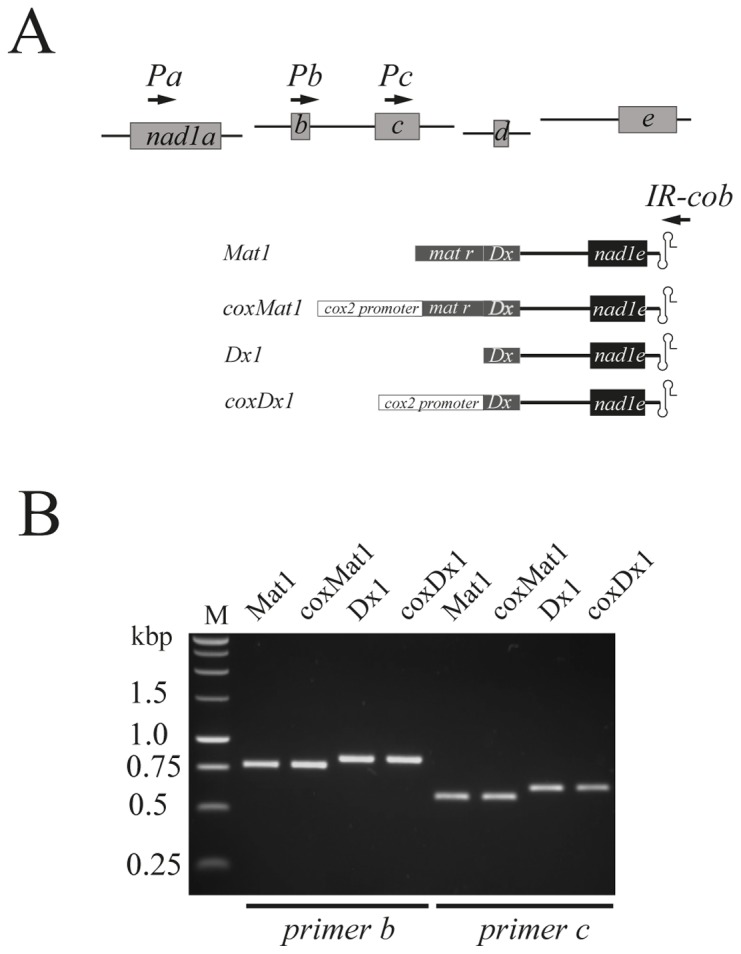
In organello *trans*-splicing after electroporation of *nad1e* recombinant vectors. (A) The four transcription units required to assemble the *nad1* mRNA in wheat mitochondria [21] are indicated in the upper part of panel A (*nad1* exons a, b, c, d, and e are represented by boxes). The four chimeric *nad1e* transgene constructs, *Mat1* containing all the 3′-half intron 4 (*3*′ *nad1-I4*) and the *nad1e* exon, *Dx1* containing only the maturase domain (Dx) from the *mat-r* ORF were linked to the inverted repeat (double stem-loop) from the non coding region of wheat apocytochrome b gene (*Ir-cob)*. These constructs were fused to the cytochrome oxidase subunit 2 (*cox2*) promoter to obtain *coxMat1* and *cox2Dx1* recombinant vectors. The arrows signal the specific exon and *cob* primers used in electroporation *trans*-splicing analyses. (B) Agarose gel electrophoresis of RT-PCR products obtained with the primer *cob* combined with primers located either in exons *nad1b* (Pb) *or nad1c* (Pc). The position of the PCR primers is indicated by arrows. M, molecular weight marker. The different constructs differ on upstream sequences from *nad1e* exon.

To verify if RNA editing play a role in the *trans-*splicing process, we introduced engineered *matr-nad1e* constructs into isolated mitochondria by electroporation [31]. Based on previous observations indicating the presence of an internal promoter in the *mat-r* ORF [24], four different constructs were designed for use in electroporation experiments (Figure 4A). Two contain either the complete *mat-r* ORF (*Mat1*) or only the maturase domain (*Dx1*), and two others, *coxMat1* and *coxDx1*, contain an additional *cox2* promoter [31]. The *in organello* expression of all four constructs produced the chimeric gene (see File S3). The constructs devoid of the *cox2* promoter showed detectable transcript products, indicating that the internal promoter from *mat-r* [24] is functional (Figure 4, lanes *Mat1* and *Dx1*).

Because the expression of two different vectors carrying the *trans*-splicing partner genes in the same compartment is hindered by the lack of selection pressure, we surveyed splicing of transgenic RNAs with cognate endogenous transcripts. PCR reactions were performed using primer *cob* combined with primers located either on *nad1a* (Pa), *nad1b* (Pb) or *nad1c* (Pc) exon (see File S1 and Figure 4A). All constructs generated chimeric spliced products comprising *nad1b*, *nad1c* and *nad1d* exons linked to *nad1e* (Figure 4B). No amplification product was detected using a primer located on the *nad1a* exon. Sequence analysis of the different PCR products indicated that the bands observed in electrophoresis gels correspond to *bona fide* splicing products from the *nad1e* containing transgene. The junctions between exons *nad1b*-*c*-*d*-*e* are comparable to the endogenous mRNA (File S4), indicating that the transgene transcript was successfully incorporated to the processed RNA pool.

### Editing Status of the Transgenic *nad1e* Exon

**Figure 5:**
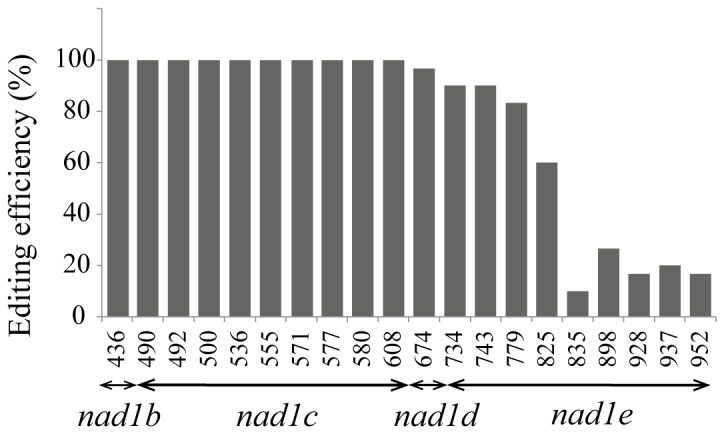
RNA editing levels within the spliced *nad1* chimeric transcript. The SDx construct was electroporated into isolated mitochondria as described in Materials and Methods. After 18 hr incubation, the mtRNA was isolated and the chimeric spliced products were obtained by RT-PCR using primers located in the exon *nad1b* and the terminator *cob*. The PCR product inserted into the pGEM-T vector was cloned and sequenced. Editing levels of C-targets on exons *nad1b* to *nad1e* represent the average of 29 independent clones (gray bars). The positions of C targets in the mature *nad1* transcript, considering the first nucleotide the beginning ATG codon from exon *nad1a,* are indicated below the bars.

C-to-U conversions generated by RNA editing of the spliced transcripts issued from *Dx1* trangene electroporation were evaluated by sequencing 30 individual PCR clones. The editing efficiency was determined as the percentage of T residues found at the place of specific C genomic residues on PCR products (Figure 5). Nine editing sites are found in the *nad1e* exon [24], and four, one, nine and one editing target Cs are present in exons a, b, c and d respectively [21]. All editing sites on *nad1b*, *nad1c* and *nad1d* exons are changed into U, while the *nad1e* exon shows a variable level of editing. More than 60% of clones were edited at sites C734, C743, C779 and C825, which are closest to exon d, whereas the five 3′ distal C-targets (after C835) were less efficiently edited. The *nad1e* exon is full edited in mature endogenous *nad1* mRNA in mock electroporated mitochondria.

### Mutation of the D6 Editing Target Residues Affects Splicing

**Figure 6:**
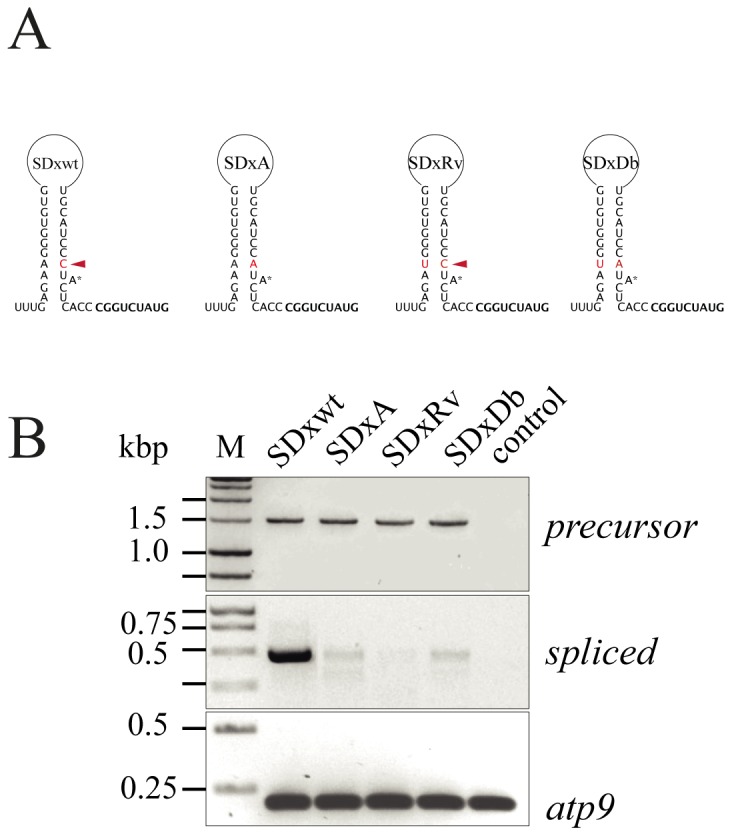
Expression levels of the chimeric *nad1e* mutants. (A) Putative hairpin structure of D6 domain from the *nad1e trans*-intron in the constructs used in these experiments. The edited version of the D6 region (SDxwt) and the SDxA, SDxRv and SDxDb mutants are presented. The arrow indicates the position of the editing target C residue in the precursor transcript. Red letters indicate the residues modified in the constructs. (B) Agarose gel electrophoresis of PCR products from *trans*-spliced *nad1*. The introduced gene contains the *nad1e* exon and the 3′-half intron under the control of the mitochondrial *cox2* promoter (for details see Materials and methods). The upper panel shows the PCR product from precursor cDNA molecules. The middle panel shows the *trans*-spliced *nad1c*/*nad1d* exons, linked to the recombinant *nad1e* exon. The lower panel shows the PCR products of the endogenous *atp9* transcripts as a control for RNA preparations from electroporated mitochondria. Only RNA samples where PCR analysis showed no DNA contamination were used for further analysis. No amplification products were observed on cDNA from untransformed mitochondria using the primers specific for the *nad1* trans-splicing analysis. The primers used are detailed in File S1. The signal in gels corresponds to one (*atp9*), two (precursor *nad1e*) or three (*trans*-spliced product) rounds of 20 PCR cycles. The sequence of precursor and spliced PCR products were determined after purification from the gel.

To verify whether the change in C-residue editing on the D6 stem of the *nad1e* precursor is important for splicing, the C target was changed to A (SDxA). Another construct where the partner residue on the complementary region was replaced by T (SDxRv) was also made, as well as a combination of both modifications in a single construct (SDxDb). As depicted in Figure 6A, SDxA and SDxRv mutants prevent the formation of the putative stem-loop structure, while the double mutant (SDxDb) restores the base pair capacity at this position. To ensure that the electroporation experiments allow a reliable comparison between different constructs, endogenous *atp9* mRNA levels were used as a control of the cDNA quality (Figure 6B, lower panel). Except for the endogenous *atp9* cDNA (primers atp9F and atp9R), no PCR signal was detected on cDNA from mitochondria electroporated in the absence of DNA (Figure 6B, lower panel) when using specific transgene *nad1* primers (File S1). The spliced products were analyzed by three rounds of nested PCR using primers Pc1, Pc2 and Pd1, combined with cob3′(1)AS, cob3′(2)AS and cob3′(3)AS for 20 cycles. The C to A mutation (SDxA) strongly reduced splicing compared to the wild type construct SDx although some product can still be detected (Figure 6B, middle panel). Densitometry analysis of the images indicates that less than 1% of spliced product was produced by mutants based on three independent experiments. A stronger decrease in splicing efficiency was observed for the SDxRv mutant, indicating that the opposite residue on the complementary strand is also important for *trans-*splicing of *nad1e*. To investigate whether the inhibition is resulting from the inability to ensure base pair formation stabilizing the D6 stem-loop, a double mutant (SDxDb) was used. In this case, the splicing efficiency was not restored to wild-type like levels, but a faint band similar to the one generated using the SDxA construct was detected. The precursor transcript levels generated by the introduced gene were analyzed using primers cob3′(1)AS, cob3′(2)AS, combined with Dx1S and Dx2S respectively, as an indicator of transgene expression. We found that all constructs were expressed at comparable levels (Figure 6B, upper panel), indicating that the varied abundance of spliced products resulted from modification of the splicing competence of mutant constructs. Precursor and *trans*-spliced cDNA correspond to genuine transgene transcript products as verified by sequence analysis.

## Discussion

RNA editing can be found in different structural domains of mitochondrial introns, but not all modifications are associated with the splicing process [3], [14], [15], [19]. Group II intron splicing involves two sequential transesterification reactions. The first is a nucleophilic attack of the 2′-OH of a bulged Adenosine residue of D6 on the 5′ splice junction, releasing the 5′ exon and generating an intron-3′ exon branched intermediate. The second reaction involves a nucleophilic attack on the 3′ splice site by the 3′-OH of the last nucleotide of the 5′-exon, yielding ligated exons and the intron RNA lariat with a 2′–5′ phosphodiester bond as the major splicing products [12], [35]. The presence of C-to-U changes in the D6 on both *nad1e-I4* and *nad5c-I2 trans*-introns from the co-transcript *mat-r-nad1e-nad5c*, raises the question of the functional significance of RNA editing in non-coding regions. The secondary structure of the D6 intron in both *nad1-I4* and *nad5-I2* is similar, albeit different in primary structure after editing (see Figure 1B). Both are predicted to adopt a canonical 2D loop-stem structure with an Adenosine residue in a bulge forming the branching point during the first transesterification step [12], [27].

To assess whether the editing events on *nad1e-I4* and *nad5c-I2 D6 domains* are necessary for *trans*-splicing of both *nad1e* and *nad5c*, we analyzed the branched molecules resulting from splicing (Figure 2 and 3). This approach allows to amplify two type of branched molecules: (1) a branched intermediate in which the second transesterification step has not yet taken place and (2) a fully-excised introns. Interestingly, a sequence survey of the D6 region indicated all the branched products presented a U residue at the editing target site, while the precursor RNAs shows a mixed pool of transcripts with either a C or U residue (Table 1). The strong bias for editing the C residues in D6 in intermediate molecules supports the hypothesis that editing of these residues was necessary to initiate the transesterification step, leading to the junction of *nad1d/nad1e* and *nad5b/nad5c* exons. The analysis of the *mat-r-nad1e-nad5c* cotranscript, proposed to be the substrate for *trans*-splicing of *nad1d/nad1e* and *nad5b/nad5c* exons, offers the possibility to survey putative splicing intermediates. The *nad5c-I2* branched products shows the presence of the Dx domain from *mat-r* and the *nad1e* exon (Figure 3), indicating that the excision of the *nad1e-I4 trans*-intron is not required for the splicing of *nad5c* and suggests that the *trans*-splicing process occurs probably at random for both, *nad1e and nad5c* exons. All these branched molecules, present the *nad5c-I2* and *nad1e-I4* editing C-targets changed to U.

We also noticed that in several cases the branched *nad1e* and *nad5c* splicing intermediates contain 3 to 8 extra residues downstream of the branch point (not shown). These results are consistent with the observations made by Li-Pook-Than and Bonen [33]. Under our assay conditions, we did not found molecules presenting random missplicing with different regions of the transcript.

To study the role of RNA editing of domain D6 from 3′-*nad1-I4 in* the *trans*-splicing process, engineered *mat-r-nad1e* transgenes were electroporated into mitochondria [36], [37]. The transgene product from the chimeric construct containing the *nad1e* exon tagged with the *IR-cob* region was faithfully spliced to their endogenous *nad1b, nad1c* and *nad1d* counterparts endogenous (Figure 4, Figure 6 and File S4). Contrary to the steady state endogenous mRNA, we fail to observe a chimeric messenger comprising the *nad1a* exon. In the model presented here, the chimeric construct is expressed throughout the eighteen hours following electroporation, compared to the steady-state endogenous *nad1* RNA pool already present in isolated mitochondria. It has been proposed that the maturation of functional mRNA through *trans*-splicing may be subject to a specific order of exon combination in wheat *nad5* [38]. One possibility is that *nad1a* is the last assembled exon, but we cannot discard that it is less efficiently spliced in the experimental conditions used.

As mentioned, *in vivo,* all the analyzed branched molecules presented the D6 editing target changed to U. Although the precise secondary structure of *nad1e* and *nad5c* D6 domains remains to be determined, after editing changes, the stem-loop structure of *nad1e* and *nad5c* D6 domains (Figure 1B) are the best one based on the similarity with the D6 region from other group II introns [25]. The bulging Adenosine that serves as the branch point during splicing is proposed to be formed in the 3′-half of the *nad1e-I4* and *nad5c-I2* introns after C-to-U editing. To prevent the reorganization of the D6 stem-loop, we changed the D6 C residue to an A, a non editable residue, which led to a dramatic reduction, but not complete, extinction of *trans*-splicing (Figure 6B). Similarly, a strong reduction of splicing level was observed in a mutant where the complementary A base on the stem was changed to a U (Figure 6A, B). The dramatic decrease of *trans*-splicing resulted from D6 sequence modifications were the result of an inefficient splicing and not an inefficient transcription since expression of the transgenic precursor was similar for each mutation and wild-type control. Surprisingly, we found that restoring the base-pairing capacity of the mutant A residue by changing the complementary A to a U (Figure 6, lane SDxDb), does not recover splicing and remained below 1% of the wild-type splicing efficiency.

While these results indicate the importance of the D6 C residue, the low *trans*-splicing levels detected in the mutant constructs indicate that, in the conditions of the experiment, inefficient transcript maturation of *nad1e* can proceed without the putative changes to the D6 stem-loop. This is different from previous results where artificially restoring the base pairing ability in either D1 or D6 domains of *cis-*introns recovers splicing competence [20], [39]. It has been reported that suppression of the branch-site of D6 in a self-splicing intron does not preclude splicing because the first step is hydrolytic instead of a transesterification [40]–[42]. Why mitochondria chose the transesterification pathway that involves RNA editing events on D6 of the intron instead of a hydrolysis mechanism remains unknown. It may be argued that the two transesterification steps occur as a coordinated process, making it more efficient than hydrolysis followed by transesterification of a free exon.

The contacts between residues stabilizing the tertiary structure of the RNA also play an important role in splicing. It was reported that domain 5 (D5) of the intron facilitates the *trans*-splicing reaction [43]. Moreover, the D6 domain is proposed to exist as two functional conformations, one participating in branching and another silent conformation that is stabilized by the η−η′ interaction between D6 and D2 [44], [45]. However, the details of the mechanisms involved in these processes are still a matter of debate [25], [35]. It has been proposed that a tertiary contact between the residues of domain VI, surrounding the branch point, with the sub domain IC1 from domain I (D1) can affect branching rather than hydrolysis reaction [25], [46], [47]. Based on these observations, one possibility is that the inability of the SDxDb transcripts to be spliced may be explained by the fact that the C residue interacts with the receptor site of D1. Another possibility is that protein factors assisting in splicing combined with the intron mutations may affect some protein-RNA interactions acting in this *in organello* approach, since the putative protein factors are already present in electroporated mitochondria. In our model, the *nad1e-nad5c* transcript is associated with the *mat-r* ORF [23]. It has been proposed that the intron-encoded maturase protein from bacterial group IIC introns may control the fate and dynamics of D6 which cannot undergo branching *in vitro* in the absence of the maturase, splicing exclusively through the hydrolytic pathway [48], [49]. However, the precise role of the putative MAT-R in mitochondrial RNA maturation, if any, has not been elucidated.

Concomitant to the RNA editing study, the *in organello* approach allows us to verify the question raised by analysis of the steady-state *mat-r-nad1e-nad5c* RNA pool [24]. Two transcripts bearing both *nad1e* and *nad5c* exons were found in mitochondria: one associated with the complete *mat-r* ORF and another, more abundant, containing the 3′ Dx domain from *mat-r* [24]. The second one was proposed to originate from an internal promoter located between the RT and maturase Dx domains. We confirmed this model since the transgenes devoid of the *cox2* promoter faithfully express the *nad1e* mRNA (Figure 4).

This study indicates that, at least for *mat-r-nad1e-nad5* transcripts, editing is important for both *trans*-splicing events considering the strong bias for a U-edited residue in the D6 region of the intermediates. In addition, this work demonstrated that it is possible to use mitochondrial electroporation to study *trans*-splicing to address different questions hardly accessible by other approaches. Indeed, the editing pattern and the splicing junctions of transgenic products reveal that we obtained *bona fide* mature products from transgenic constructs (Figures 5 and File S4), opening the way to unveil problems raised by this complex mode of expression.

Considered together, our results show that group II introns in plant mitochondria use a variety of strategies to assist splicing, and support the hypothesis that RNA editing contributes to an RNA structure amenable to splicing.

Interestingly, RNA editing is a very abundant process in non-coding regions in mammals [50], [51] and recently, RNA editing within an intron has been implicated in the regulation of alternative *cis*-splicing [52]. In addition, *trans*-splicing has also been described in mammalian cells [53], but the involvement of RNA editing in this process remains to be discovered.

## Supporting Information

File S1 **Primers used in this work.**
(PDF)

File S2 ***Triticum aestivum*** **mitochondrial NADH dehydrogenase subunit I (** ***nad1*** **) and subunit 5 (** ***nad5*** **) genes involved in** ***trans*** **-splicing. (A)** Wheat mitochondrial NADH *nad1* gene, exon 4 (ACCESSION X57966) and transcript unit for *mat- r-nad1e-nad5c* (ACCESSION X57965. (Chapdelaine and Bonen (1991) Cell 65∶465–472). The 3′ half *nad1-I4 trans*-intron containing the Maturase- Reverse Transcriptase (mat-r) reading frame (2134 nt) is underlined. The downstream region (grey letters)is formed by the 3′-half *nad5-I2* intron, the *nad5c* exon and the 5′-half *nad5-I3* intron. The position of primers (P1a, P1b, P2a, P2b, P3a and P3b) used to analyze the branched splicing intermediates are blue highlighted. Exons regions are indicated in red (*nad1d* and *nad5e*) or black (*nad5c*) uppercase letters. C-editing targets throughout the sequence are indicated as red lowercase letters. Ten nucleotides in underlined italics bold letters (yellow highlighted) indicate the limits of the sequences from the co-transcript used for recombinant *Mat1*, Dx1 and their derivatives used in electroporation experiments. Details on the assembling procedure are described in the Methods section. **(B)** Sequence of the wheat *nad5* scatterd gene **(**GenBank: AH001278) **(**Pereira de Souza et al. Plant Cell 3 (12), 1363–1378 (1991). Only the transcript unit containing exons *nad5a* and *nad5b* is presented. The downstream region from *nad5b,* containing the 5′-half of the *trans*-intron *nad5-I2,* is part of the co-transcript *mat-r- nad1e-nad5c* (see above). The position of primers P1b and P2B used to analyze the *nad5b/nad5c* branched splicing intermediate are blue highlighted.
(PDF)

File S3 **Expression of** ***nad1e*** **transgenes after electroporation of isolated wheat mito- chondria.** (A) Four constructs were used: (*Mat1*) contains all the 3′-half intron 4 (*3′ nad1-I4*) and the nad1e exon, linked to the inverted repeat from the non coding region of wheat apocytochrome b (*cob*) gene; (*Dx1*) contains only the domain maturase (*Dx*) from the *mat-r* ORF was linked to the *Ir-cob* region. The same sequences were fused to the cytochrome oxidase subunit 2 (*cox2*) promoter to obtain the recombinant vectors *coxMat1* and *cox2Dx1*. (B) Agarose gel electrophoresis of PCR products obtained after three nested PCR reactions of 20 cycles each, using primers cob3’(1)AS, cob3’(2)AS or cob3’(3)AS, combined with primers Dx1S, Dx2S or Dx3S. One of the four PCR control reactions made on samples where reverse transcriptase was omitted in cDNA reactions is shown (-RT). The DNA size marker BenchTop 1kb DNA Ladder (Promega). Primers used are indi- cated in File S1.
(PDF)

File S4 **Electropherogram from a cloned** ***nad1 trans*** **-spliced PCR product obtained after electroporation of the** ***nad1e*** **chimeric gene.** (A) The junctions between exons *nad1b* and *nad1c*, the complete exon d, and the junctions with *nad1c* and the fusion between exons *nad1c* and *nad1e* are shown. (B) Sequence of the 3″end of the chimeric transcript showing the link between the end of *nad1e* and the *IR-cob* terminator sequence.
(PDF)
