# Supplementary material for: RNA Editing in Mitochondrial Trans-Introns Is Required for Splicing
Source: PLoS One. 2012 Dec 20;7(12):e52644. doi: 10.1371/journal.pone.0052644 (PMC3527595; doi:10.1371/journal.pone.0052644)
Supplement: File S1 — Primers used in this work. (PDF) [file pone.0052644.s001.pdf]

Supplemental S1. Primers used in this work. (only sens primers are indicated)

Primers used to study the splicing branched intermediates

P1a	CACACTAGGCTACCCTTCTCCG
P2a	GCCGGAGGGGTTTATTGCACA
P3a	AGGCCCGACGAGCGGTCCACG
P1b	TTATGACGGTCACCAGGGCC
P2b	ATTGTGACCTCGTACGATCGT
P3b	GCTCCACGTACGGTTTTGAAG
P4	GACCAACGTCAGCGACAGAG
P5	TTGGCTGCCTATCCTAGATCT
P6	TACCTAAACCAATCATCATATC

Primers used for the endogenous transcript (cDNA analysis):

atp9F	ATGTTAGAAGGTGCTAAATC
atp9R	CGATCGCATAAAAAGTCATGA

Primers used for the analysis of the transplicing products:

Pa1	GTGCTAGCTGAACGTAAAGTAATGGCTTTTG
Pa2	TTGTGCAACGTCGAAAGGGT
Pa3	GATGTAGTGGGATCGTTC
Pb1	CGAAATATGCCTTTCTAGGAGCATTACGATCTG
Pb2	AGCTCAAATGGTCtCTTATGAAG
Pb3	ctctattggtcttattcttattg
Pc1	GTCTAGCAGAAACTAATCGAGCTCCGT
Pc2	CTCCCAGAAGCGGAAGC
Pc3	GAATTAGTTGCAGGCTATAAT
Pd1	ATGTAGAATATTCTTCAATG
cob3'(1)AS	AGCCCTCATACCCACTCCTC
cob3'(2)AS	TCCCGCGGGAAGCGGAAAGC
cob3'(3)AS	TATCCAGATTTGGTACCAAACCC
Dx1S	TGTCCTGAAGAAGAAATTGTATACTCCCTT
DX2S	GGGATCTTTCAGCAGCGAATT

Primers used for *cloning mat-r-nad1e* constructs. Restriction sites are underlined:

Sac-MatR	atctgagagctccttccttaaagagcctttcg
Sac-Dx	atctgagagctcgactcttggaaaagatggac
Nsi-MatR	atctgaatgcatcttccttaaagagcctttcg
Nsi-Dx	atctgaatgcatgactcttggaaaagatggac

SmaI-ex5nad1 atctgacccgggtcctgcccgcagccctattc
Spe-ex5nad1 atctgaactagttcctgcccgcagccctattc

Primers used for *site directed mutagenesis of the editing residue on the D6 of nad1e-I4*. Low-case letter indicate the modified residue:

mutCtoA-D6nad1-S GGAGCTGCATCCaTACTCACCCGGT
mutAtoT-revD6-S GTTCCTTTGAGAtGGGTGTGATACC
