# Supplementary material for: RNA Editing in Mitochondrial Trans-Introns Is Required for Splicing
Source: PLoS One. 2012 Dec 20;7(12):e52644. doi: 10.1371/journal.pone.0052644 (PMC3527595; doi:10.1371/journal.pone.0052644)
Supplement: File S2 — Triticum aestivum mitochondrial NADH dehydrogenase subunit I ( nad1 ) and subunit 5 ( nad5 ) genes involved in trans -splicing. (A) Wheat mitochondrial NADH nad1 gene, exon 4 (ACCESSION X57966) and transcript unit for mat- r-nad1e-nad5c (ACCESSION X57965. (Chapdelaine and Bonen (1991) Cell 65∶465–472). The 3′ half nad1-I4 trans-intron containing the Maturase- Reverse Transcriptase (mat-r) reading frame (2134 nt) is underlined. The downstream region (grey letters)is formed by the 3′-half nad5-I2 intron, the nad5c exon and the 5′-half nad5-I3 intron. The position of primers (P1a, P1b, P2a, P2b, P3a and P3b) used to analyze the branched splicing intermediates are blue highlighted. Exons regions are indicated in red (nad1d and nad5e) or black (nad5c) uppercase letters. C-editing targets throughout the sequence are indicated as red lowercase letters. Ten nucleotides in underlined italics bold letters (yellow highlighted) indicate the limits of the sequences from the co-transcript used for recombinant Mat1, Dx1 and their derivatives used in electroporation experiments. Details on the assembling procedure are described in the Methods section. (B) Sequence of the wheat nad5 scatterd gene (GenBank: AH001278) (Pereira de Souza et al. Plant Cell 3 (12), 1363–1378 (1991). Only the transcript unit containing exons nad5a and nad5b is presented. The downstream region from nad5b, containing the 5′-half of the trans-intron nad5-I2, is part of the co-transcript mat-r- nad1e-nad5c (see above). The position of primers P1b and P2B used to analyze the nad5b/nad5c branched splicing intermediate are blue highlighted. (PDF) [file pone.0052644.s002.pdf]

SUPPLEMENTAL S2. *Triticum aestivum* mitochondrial NADH dehydrogenase subunit I (nad1) and subunit 5 (nad5) genes involved in trans-splicing

(A) *Triticum aestivum* mitochondrial *nad1* gene, exon 4 (ACCESSION X57966) and transcript unit for *mat-r-nad1e-nad5c* (ACCESSION X57965. (Chapdelaine and Bonen (1991) Cell 65:465-472).

The 3' half *nad1-I4 trans*-intron containing the Maturase-Reverse Transcriptase (mat-r) reading frame (2134 nt) is underlined. The downstream region (grey letters)is formed by the 3'-half *nad5-I2* intron, the *nad5c* exon and the 5'-half *nad5-I3* intron. The position of primers (P1a, P1b, P2a, P2b, P3a and P3b) used to analyze the branched splicing intermediates are blue highlighted. Exons regions are indicated in red (*nad1d* and *nad5e*) or black (*nad5c*) uppercase letters. C-editing targets throughout the sequence are indicated as red lowercase letters. Ten nucleotides in underlined italics bold letters (yellow highlighted) indicate the limits of the sequences from the co-transcript used for recombinant *Mat1*, Dx1 and their derivatives used in electroporation experiments. Details on the assembling procedure are described in the Methods section.

(a) Triticum aestivum mitochondrial *nad1* gene exon 4 (*nad1d*) transcrption unit.

ctcgagaccgctctccgtccatatttaggacggttagggcgatttttaagaaataaaaaccttctgt
cgatttatccacacttccatgacttctagaggaaagctaactgcttgctggctgggagctgtatgagc
ggtaacgtccacgtacggctccgtgagaaggtggacggaaatggccttgttgtacctcactcccgTCT
TCAATGGGGTCTGCTCTTTTTTTTTTAGGAGAGTATGCCAATATGATCTTCAATGAGgtgcggggctt
tgcatctgacattcgttgggctttcctctgcgggagcccgcgtcccggcctttttgtgcaataaaccc
ctccggccgaagactagtgataggtggtcccgcggagctttcggagaagggtagcctagtgtgtaagc
acagcaatgaaccgcggcgaaccctcagacgacccttctaagataaggggggagatcctcagtagtgg
tgaccctttgactcttccactgacttatatatgtaccgaatgctcatacgggaaagtgaactcctggg
tctggaacctgggggggttgctccgataaaaaatcctttctttctcgtccactctagggggtgcggac
acacctgcgcggattacaggtgacggttacaagaatggcggggaagtgaagagtacccgacgacattc
agggatgaatgtagacccatcgggcggggataatcattccggtcctgggagaggtggcgacaccagtc
tgagctgagggaagccagccaattgagtcactgaaacgaccgcaggaggcgcaccctaagcccagctt
ctcggagctgctattgcgaaatacg

(b) *Triticum aestivum* mitochondrial co-transcript containing mat-r, *nad1e* and *nad5c* and the 3'half introns *nad1-I4* and *nad5-I2*.

cccgggtccaaacctgctgagctagctaatttttcctttacgagatctcggctcttcggaatgattgg
agagagccccgcctcctcttggttggtgccgggcccgagagtgatgggacta***cttccttaaa***gagcct
ttcgttcgggccggcaggagggccctgatctatcaattgagggagcaaaaaacaggctttgctcccc
ctttttaaatgaagaaagaaagaagggtcgaagtttagaccgctcacagtagttctacccatagaaa
agatcatgaaagaggcgatcagaatggtacccgaatccatttacgatcccgagtttccagacacatcg
cacttccgctcgggtcgaggctgccactcggccctcagacggatcaaagaagagtggggaacctctcg
ctggtttttggaattcgacatcaggaagtgttttcacaccatcgaccgacatcgattcatctcaatct
tgaaggaagagatcgacgattccaagttcttttaccccactcagaaaaagttttccgccggacgactc
gtaggggggtgagaagggccctgactccatcccaaacagtgtactactatcggccctaccaggcaatat
ctacctacacaagctcgatcaggagatagggaggatccggcagaagcacgaaattcctcttgtagtga
aaataagatcggttctattaagaataggtcgtcgtattgatgaccaagaaaagtatggaaaagaagca
agcttcaacgctccccaagacaacagagccctcatagtggggagggtaaagagcatccaacgcaaagc
gacctttcattcccttgtttcgtcgtggcacaccccccccacaagcacccccaggcgaaggggagacc
agaaaacgcctttcgtttttccccccttcagcggccctagccgccttccttaacaagccctcgagcctc

ctttgcgccgccttcctcatagaagccgctgggttgaccccgaaggccgaattcaatggtagagaagg
ctttaataagaatttggccatgagagaccttcttaagtattgcaaaagaaggggcctgctgatagagc
tgggcggggaggcgatactagttatcaggtcagagagaggcctggcccgtaagctggcccctttaaa
agccattccttattaataaggatttgttacgcgcgatatgccgacgacttactactgggaatcgtggg
tgccgtatttcttctcatagaaatacaaaaacgtatcacccacttcctacaatccggtctgaaccttt
gggtaggctccgcaggatcaacaaccatagctgcacggagtacggtagaattccccggtacggtcatt
cgggaagtcccccgaggacgactcccatacaattcttgcgagagctggagaagcgtctacgggtaaa
gcaccgtatccatataactgcctgccacctacgctccgccatccattccaagtttaggacctaggtt
atagtatccctatcaaagagcttacgaaggggatgagcggaagaggtcgtctactggacgcggttcaa
ctagcggagactcttggaaaagatggactaaaaagtccccaagttagcgtat***tatggggac***cgtcaa
gcacatccggcaaagatcgaggggatctcgttgttgcatagctcaggtcagagcaaggtgccatcag
gcgttcaacaggcagtctcacgatcgggcatgagtgtcctgaagaagaaattgtatactccctttggt
cggaaggcggcgggggaaggaaggggacactgggcgggatctttcagcagcgaattccccatacagat
agaggcgcctatcaaaaagatactccgaaggcttcgggatcgaggtctcattagccgaagaagaccca
ggccaatccacgtggcctctttgaccaacgtcagcgacagagacatagtaaattggtccgcgggcatc
gcgataagtcctctgtcctactacaggtgctgcgacaacctttaccaagtccgaacgattgtcaacta
ccagatccgctggtccgctatattcaccctagcccacaagcacaaatcttcggcgcggaatataatcc
taaagtaccccaaagactcaaatatagtcaatcaagaaggtggtaagacccttgctgagttcccaaac
agcatagagcttgggaagctcggactcggtcaagatccgaacaacgacggagcactcaactacatgtt
taataagtagcgatttagcgaacaggcgtttacatgagattagttgagtaggcttgcttatctgcta
taagatagctagttgtggggctttcgaaaaaaaaaaggctgaaggcttcgctatcgctcatggcttgt
attgtagtcgtagtcttgtagtcggccctccatgcctctttagtctttctaatcctgaggccttttc
cttcattcattttgcggtagttacgcgtcagaaaaatagaacctttccggcccggaagatcgctgctt
cgcttctggcgactagcttctacccacaatggctaaagctaccttgaatcaatcaatgaatgaatgat
tcgtaacccccatgggttcgaggacccgttggtcaaaggaaaagggggtccagattccaggacggag
ccgtatgaggcgagagtctcacgtacggttcctttgagaagggtgtgataccaccacctatcaggccc
gacgagcggtccacggagctgcatccctactcaccCGGTCTATGCACATcGCTCTTTCcAGGAGGTTG
GCTGCCTATCCTAGATCTTCCCATTTcCAAAAAGATCCCTTGTTCGATCTGGTTTAGTATCAAGGTTC
TTcTTTTTCTGTTTcTATATATATGGGTCCGTGCAGCATTTCCACGATATCGTTATGATCAATTAATG
GGACTTGGCcGGAAAGTGTTCTTGCCTCTATCATTAGCTcGGGTAGTCcCCGTTTCAGGTGTTTcAGT
CACCTTTCGATGGCTCCCTTAAtgtatgtgccaggaagtttcttctgaaggggctactccccgaaaa
tgccccgccccttgttcgtttgtcgttggggattcattgctcgttctgcggttataagtaacgtagcc
agcctaatttccagaatagggct***gcgggcagga***gggctttgtttgtgcaatcttgctcgcaacaccct
ctcctccggcgaacctgcgatcgccgcctctaactgaatgctcaactgaggtcgtgtacaacaacctt
aaccgcacaagcctgggctgggcctacccccatccctagaggagccgtatgaggcggaagctccacgt
acggttttgaagccgagcctttccagcaatggggcctagggaccGATATGATGATTGGTTTAGGTAgg
gcggccggcctactatgggcacctgtagggattagtgcgtgagaccgcgatccacaaactgacgcatg
ggactcacccttgacttgagaatggaagaagggaaacatagcatgtcacaagagcgaggcgaggggct
ccgccctacagcgagagggacgcctcgcgagccgggctttggagatgaggccttttcgcgaagccaag
tcaatttcgggccaccaaaccctgcaactgaggaaaaggccctatggagtaaagggaagcgtgtacgt
tgtcacactccccgccctccaaaggtgcctagaggacgggccagacgcagcagagcgacgccccagga
gcggattccccacgagcaggggggacaggagacggccatctcgaggcacatcacgacctacaggcaaca
ccggcgagacccaggaaggtaacccgattgggagtcagaggatccatagtacccgcagcctcccggac
ttcatattcatcattttctaaagaaagggtaaggatctcttttctgcaacggaaaaaaaataagctc
cgctgatttgactcggcacaacctaacgatacatccaataccaatgatctgtgcctaccgatacgata
ctcctccaaaaaattgactatataaagag

(B) Sequence of the *T. aestivum nad5* scatterd gene (GenBank: AH001278) (Pereira de Souza et al. Plant Cell 3 (12), 1363-1378 (1991). Only the transcript unit containing exons *nad5a* and *nad5b* is presented. The downstream region from *nad5b,* containing the 5'-half of the *trans*-intron *nad5-I2,* is part of the co-transcript *mat-r-nad1e-nad5c* (see above). The position of primers P1b and P2B used to analyze the *nad5b/nad5c* branched splicing intermediate are blue highlighted.

gttatttcgtacaggctattcaaggtctattggctttattgcttatgtggtacttcgaccgcgaagcc
tcgcttatgcaccgagaaagatcgtagataggaaagatctgttatgcaccaacgacggccccttctct
ttacctttatcgtctcatacctcagtccattgctggcttgaatgctgtctctcttcttctttgctcgc
gagagtgaataagaggacgacccaccgcggggaggatggaatgagtcggcaaggggatcaaccatctt
ctttcagtaaaggcccacacattatccttaatattgggataaaaggactctgaagaaggaaactctct
tcccgtcttgcgtaactgaccactgcaaatcaagtcgaaagtgcttatatgcttaacacgtttttgtc
tcaatgacaggccgcttgaacattgtctgatttttttttaagagactcgatcttATGTATCTACTTAT
TGTCTTTTTGCCTTTGCTCGGTAGTTCCGTAGCCGGTTTTTTCGGACGTTTTCTAGGATCTGAAGGAA
CCGCTATAATGACCACCACGTGCGTTTCATTTTCTTCGATCTTATCTTTGATTGCTTTTTATGAAGTC
GCACTGGGAGCTAGTGCTTGCTATCTCAGAATAGCTCCATGGATCTCATCGGAAATGTTTGATGCTTC
TTGGGGCTTCTTtggcgaccgtgaagtcaccggatgaattgccgagtagatagatcagatccggacgc
tgcagttgttccgcggtgatacggactcgacccgctcctacccaccctggggtatcatagcatgtcgg
gaatcgagggggggacatactggacgtaactactcccgtcggttgggggctctgccgccctgcctttcg
atcgatacacagttgaggaggccgatcacgaacgtgacaggtgtgggagcgatcctgggaaggcaagg
ctaagacggcgccttgcatatgggtagcaagagggcgcttatgccccgacggtggggccttatgggga
agggcccagcccaatagggacagcacacccccccactttaagcgcatctctgtatcgactgaataactc
tatctaagggtgacgtcggtggaaccggtgaaccacgcgagctggttagatgcgtggggcagagggct
cgtagtacccccctttcttgatccagccttttcttcgcttcggtagttaatcacctaaaatccaagg
gaggctggcctgcacgccatacctatacccatacagtgccaggcaggcggtggactcattttggatt
agggaagggaagaaggggcctaagcacggcagatgccgtacacttgagtggcaaaggaaagcgagacc
gtacttcttttgccaggcctgttcttacataaggttcccgcagaagatcaagttggtgagccgtgtga
tgggaaaccttcccgcacggttcggagagcactgaagacgaatgagaggttcaccaccacatcattgc
aaggggagctcgctcgattcgcagattggcctgactcgtaattcacttttgactctgtGTTCGATAGC
CTGACCGTAGTGATGTTAATTGTGGTTACATTCATAAGTAGCTTGGTCCATCTTTATTCCATTTCATA
TATGTCTGAGGATCCGCATAGCCCTCGATTTATGTGTTATTTATCCATTTTTACTTTTTTTATGCTAATGTTGGT
GACTGGAGATAACTTTCTTCAATTATTCCTGGGATGGGAGGGAGTAGGTCTTGCTTCATATTTGTTAATTCATTTCTGGTTTA
CACGACTTCAGGCGGATAAAGCTGCTATAAAAGCTATGCTTGTCAATCGAGTAGGTGATTTTGGATTAGCTCTTGGGATTTTT
GGTTGTTTTACTCTCTTTCAAACAGTAGACTTTTCAACCATTTTTGCTTGTGCTAGTGCTCCCAGAAATGAATGGATTTTTTG
CAATATGAGATTGAATGCCATAACTCTGATTTGTATTTTACTTTTTATTGGTGCTGTTGGGAAATCTGCACAGATAGGATTGC
ATACTTGGTTACCCGATGCAATGGAGGGTCCCACTCCAGTATCTGCTTTGATTCATGCAGCTACTATGGTCACTGCTGGCGTT
TTCATGATAGCAAGGTGCTCCCCTTTATTTGAATACTCACCTACGGCTTTGATTGTTATTACTTTTGCAGGAGCTATGACGTC
ATTCCTTGCGGCAACCACTGGAATATTACAGAACGATCTAAAGAGGGTCATAGCTTATTCAACTTGCAGTCAATTAGGCTATA
TGATCTTTGCTTGCGGCATCTCTAACTATTCGGTTAGCGTCTTTCACTTAATGAATCACGCGTTTTTCAAAGCATTACTCTTC
CTGAGTGCGGGTTCGGTGATTCATGCCATGTCGGATGAGCAAGATATGCGGAAGATGGGGGGGCTTGCCTCCTCCTTTCCTTT
GACCTATGCCATGATGCTCATGGGCAGCTTATCTCTTATTGGATTTCCTTTTCTAACAGGATTTTATTCTAAAGATGTGATCT
TAGAGCTCGCTTACACAAAGTATACCATCAGTGGGAACTTTGCTTTCTGGTTGGGAAGTGTCTCTGTCCTTTTCACTTCTTAT
TACTCCTTTCGTTTACTATTTCTAACATTTCTAGTACCAACTAATTCATTCGGGCGAGACAGATTACGATGTCATGATGCGCC
CATTCCTATGGCCATTCCTTTAATACTTTTGGCTCTCGGGAGTCTCTTTGTAGGATACTTGGCCAAAgtgtgacctgttagcc
cataagtcagtactgtgacgaagcggctgttgctcacccgatacgatcgtacgaggtcacaatttacccaacacgatcatccg
gggtgaacaagaattggggatcggatgcgggcgaaattcaccgccaatggctgagatgttcagtcgactccctccccctttgt
gggggtctggacccctacgagtgagcagaaaagggaggaggaaagaggccctggtgaaccgtcataataagtgaacaagtgta
agctttgctgcccgacagtatggagtactgaccacaccgagggacaggccctgaagcgaaggacaggaacgagcggaatcaat
gtgttccaatttctggccttgcaccgaccatccaacggaccatggactaaacggccactgcctgaaaggactatgctatgtcc
aggggaccgctgccctccacaaggtacatcttgcacctatgggccgctataactatccaagaagacactcgaaaaaggaagga
taaacaaacctacccggtggactgccgagctacaagtcctacgacacaggagcgggattctctaaaaagccaaggtcttaggt
ggccaagagggcccacttggagacttgggatctcagcaggaaatgtgaaggttgtttaaagccggccgataggcgaaagagaa
tccaaaagttttgttctgatctgagtaggctcaacatagggaataccctaaccctgggaaccggggcatataaatccgcttat
ggcattcacccaagcatacagagacagatggaatgaaagaaagaaaaagtactccgcagc
