# Supplementary material for: RNA Editing in Mitochondrial Trans-Introns Is Required for Splicing
Source: PLoS One. 2012 Dec 20;7(12):e52644. doi: 10.1371/journal.pone.0052644 (PMC3527595; doi:10.1371/journal.pone.0052644)
Supplement: File S3 — Expression of nad1e transgenes after electroporation of isolated wheat mito- chondria. (A) Four constructs were used: (Mat1) contains all the 3′-half intron 4 (3′ nad1-I4) and the nad1e exon, linked to the inverted repeat from the non coding region of wheat apocytochrome b (cob) gene; (Dx1) contains only the domain maturase (Dx) from the mat-r ORF was linked to the Ir-cob region. The same sequences were fused to the cytochrome oxidase subunit 2 (cox2) promoter to obtain the recombinant vectors coxMat1 and cox2Dx1. (B) Agarose gel electrophoresis of PCR products obtained after three nested PCR reactions of 20 cycles each, using primers cob3’(1)AS, cob3’(2)AS or cob3’(3)AS, combined with primers Dx1S, Dx2S or Dx3S. One of the four PCR control reactions made on samples where reverse transcriptase was omitted in cDNA reactions is shown (-RT). The DNA size marker BenchTop 1kb DNA Ladder (Promega). Primers used are indi- cated in File S1. (PDF) [file pone.0052644.s003.pdf]

A

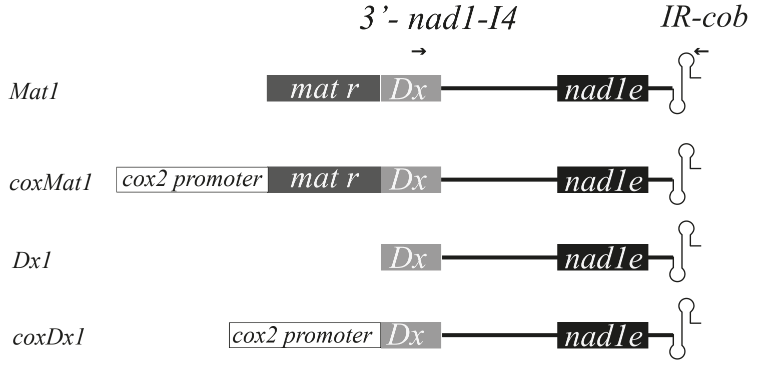

B

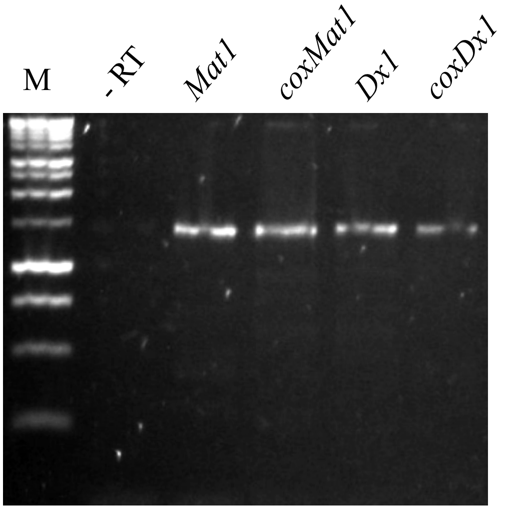

Supplemental S3. Expression of *nad1e* transgenes after electroporation of isolated wheat mitochondria. (A) Four constructs were used: (*Mat1*) contains all the 3'-half intron 4 (*3' nad1-I4*) and the nad1e exon, linked to the inverted repeat from the non coding region of wheat apocytochrome b (*cob*) gene; (*Dx1*) contains only the domain maturase (*Dx*) from the *mat-r* ORF was linked to the *Ir-cob* region. The same sequences were fused to the cytochrome oxidase subunit 2 (*cox2*) promoter to obtain the recombinant vectors *coxMat1* and *cox2Dx1*. (B) Agarose gel electrophoresis of PCR products obtained after three nested PCR reactions of 20 cycles each, using primers cob3'(1)AS, cob3'(2)AS or cob3'(3)AS, combined with primers Dx1S, Dx2S or Dx3S. One of the four PCR control reactions made on samples where reverse transcriptase was omitted in cDNA reactions is shown (-RT). The DNA size marker BenchTop 1kb DNA Ladder (Promega). Primers used are indicated in Supplemental S1.
