# Supplementary material for: RNA Editing in Mitochondrial Trans-Introns Is Required for Splicing
Source: PLoS One. 2012 Dec 20;7(12):e52644. doi: 10.1371/journal.pone.0052644 (PMC3527595; doi:10.1371/journal.pone.0052644)
Supplement: File S4 — Electropherogram from a cloned nad1 trans -spliced PCR product obtained after electroporation of the nad1e chimeric gene. (A) The junctions between exons nad1b and nad1c, the complete exon d, and the junctions with nad1c and the fusion between exons nad1c and nad1e are shown. (B) Sequence of the 3″end of the chimeric transcript showing the link between the end of nad1e and the IR-cob terminator sequence. (PDF) [file pone.0052644.s004.pdf]

A

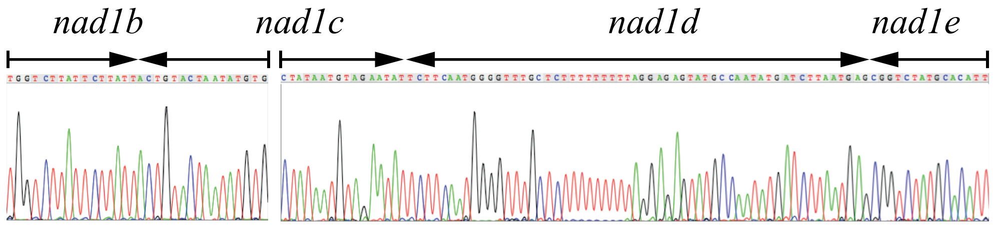

B

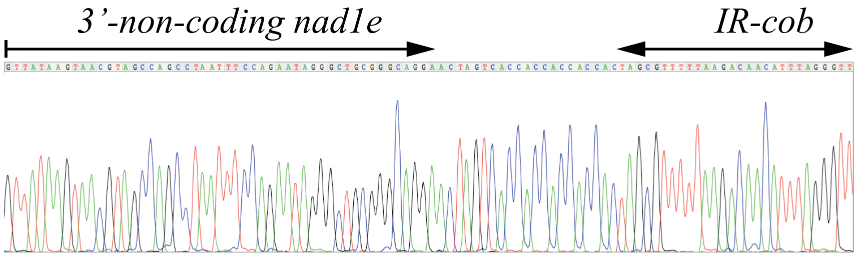

Supplemental S4. Electropherogram from a cloned *nad1 trans*-spliced PCR product obtained after electroporation of the *nad1e* chimeric gene. (A) The junctions between exons *nad1b* and *nad1c*, the complete exon d, and the junctions with *nad1c* and the fusion between exons *nad1c* and *nad1e* are shown. (B) Sequence of the 3' end of the chimeric transcript showing the link between the end of *nad1e* and the *IR-cob* terminator sequence.
